# Dietary index for gut microbiota and its protective role against kidney stones: evidence of diabetes as a mediator from NHANES cross-sectional data

**DOI:** 10.3389/fnut.2025.1532313

**Published:** 2025-02-11

**Authors:** Lei Wang, Junjun Wu, Ziwen Jiang, Chao Wang, Fuxiang Lin, Yuxiang Zhong, Pengpeng Zhao, Wei Wei, Jianhua Huang, Zhanping Xu

**Affiliations:** ^1^The Eighth Clinical Medical College of Guangzhou University of Traditional Chinese Medicine, Foshan, China; ^2^Foshan Maternal and Child Health Center, Foshan, China; ^3^School of Traditional Chinese Medicine, Jinan University, Guangzhou, China

**Keywords:** kidney stone, NHANES, microbiota, diabetes, cross-sectional study

## Abstract

**Background:**

The dietary index for gut microbiota (DI-GM) reflects dietary patterns that support gut microbial health and may influence kidney stone (KS) risk. The role of DI-GM and its mediation by diabetes in KS pathogenesis remains unclear.

**Objective:**

To investigate the association between DI-GM and KS prevalence, assess the mediation effect of diabetes, and explore subgroup-specific effects and underlying mechanisms.

**Methods:**

A cross-sectional analysis of NHANES (2007–2018) data was conducted using a stratified, multistage probability sampling design. A total of 19,841 participants were included in the final analysis. Data entry and statistical analysis were performed using Empower version 4.2 (X&Y Solutions, Inc., Boston, MA, United States) and R version 3.4.3 (R Foundation). Multivariable logistic regression was employed to assess the association between DI-GM and KS prevalence, with statistical significance set at *p* < 0.05. Mediation analysis evaluated diabetes’s contribution to this relationship, and subgroup analyses were conducted based on sex, race/ethnicity, and alcohol consumption.

**Results:**

Higher DI-GM scores were associated with lower KS prevalence (adjusted OR: 0.78, 95% CI: 0.65–0.92 per SD increase). Diabetes mediated 9.27% of this relationship. Subgroup analyses revealed stronger protective effects among females, non-Hispanic Black individuals, and heavy drinkers. Mechanistically, DI-GM may reduce KS risk through gut microbial modulation of oxalate metabolism, urinary citrate excretion, and systemic inflammation.

**Conclusion and recommendations:**

Higher DI-GM scores are associated with reduced KS prevalence, partially mediated by diabetes. These findings highlight the role of dietary interventions targeting gut microbiota in KS prevention and call for longitudinal studies to confirm these results and develop tailored dietary strategies.

## 1 Introduction

Kidney stone (KS) is one of the most common urinary system illnesses, with incidence rates increasing worldwide (1–3). The prevalence of KS varies significantly by region, ranging from 1 to 19% in Asia, about 4% in South America, and 5 to 10% in Europe (1, 4). These differences may be linked to dietary habits, genetic factors, and environmental influences that affect KS rates across diverse geographical areas (5). KS can cause severe complications, including intense pain, urinary obstruction, hematuria, and infection, which may lead to renal dysfunction and impair quality of life (6). It also has a high recurrence rate, with nearly 50% of individuals experiencing a relapse (7), which increases the risk of chronic kidney disease and necessitates substantial healthcare resources (8, 9). In the United States, for example, the annual cost of KS treatment exceeds $2 billion, highlighting its considerable economic impact (10).

Globally, non-communicable diseases (NCDs) account for 74% of all deaths annually, according to the World Health Organization (WHO). Conditions such as cardiovascular diseases, cancers, chronic respiratory diseases, and diabetes share common risk factors with KS, including poor dietary habits, obesity, and metabolic dysfunction (11). The Global Burden of Diseases (GBD) study highlights the rising prevalence of KS in middle-and high-SDI (Socio-Demographic Index) countries, emphasizing the role of dietary and metabolic factors (12). In China, a nationwide survey reported a prevalence of 5.8%, with higher rates in rural and southern regions, while in the United States, KS affects approximately 2.4% of the adult population, incurring significant healthcare costs (1, 10).

Dietary factors play a critical role in KS development and recurrence (13), as they directly influence urine composition, pH, and gut microbiota, all of which affect KS formation (14, 15). Gut microbiota imbalances can increase the production of stone-forming components, heightening KS risk. Consequently, optimizing diet to regulate gut microbiota is a promising preventive strategy. In 2016, Kase et al. (16) introduced the dietary index of the gut microbiota (DI-GM), a tool designed to evaluate diet quality in relation to gut health. This index considers key dietary components, including fiber, polyunsaturated fatty acids, and sugars, which either support beneficial bacteria or suppress pathogenic ones. By quantifying the effects of these dietary elements, the DI-GM categorizes them into beneficial and unfavorable groups. The overall score, calculated through a specific formula, reflects the diet’s favorability for gut microbiota, with higher scores indicating a more gut-friendly diet. The validity of this scoring system has been demonstrated through biomarkers such as microbial diversity indices and short-chain fatty acid (SCFA) production, which reliably indicate dietary contributions to gut flora diversity.

Diabetes is another critical factor associated with KS, potentially due to hyperglycemia, metabolic dysregulation, and renal impairment (17, 18). Diabetic patients often exhibit elevated uric acid levels, a known risk factor for chronic kidney disease and KS (19, 20). Urate stones are particularly prevalent in diabetic individuals, likely due to insulin resistance and associated metabolic dysfunction (21). However, the mediating role of diabetes in the relationship between DI-GM and KS remains unclear (22, 23).

This study leverages data from the US National Health and Nutrition Examination Survey (NHANES) (2007–2018) to explore the potential association between DI-GM and KS and examine diabetes as a mediating factor. By investigating the interactions among dietary patterns, gut microbiota, and diabetes, this research aims to provide insights into KS prevention and management strategies while aligning with global health priorities identified by WHO, CDC, and GBD studies.

## 2 Methods

### 2.1 Study setting and period

The data were sourced from NHANES (2007–2018), a nationwide survey representative of the U.S. population. Data were collected through household interviews and mobile examination centers, covering dietary intake, health conditions, and lifestyle information.

### 2.2 Source population

The source population comprised participants from the NHANES database, which aims to assess the health and nutritional status of individuals across various age groups and ethnicities in the United States.

### 2.3 Study population

Based on inclusion and exclusion criteria, a total of 19,841 eligible participants were included in the final analysis: exclusions included pregnant women, individuals with missing data (e.g., poverty income ratio, alcohol consumption, and BMI), and those with unclear KS status.

### 2.4 Variables (dependent and independent variables)

The dependent variable in this study is the presence of kidney stones (KS), determined based on participants’ responses to the question, “Has a doctor ever diagnosed you with kidney stones?” A “yes” response indicates the presence of KS, while a “no” response indicates its absence.

The DI-GM score is based on the intake of 14 specific foods or nutrients. Avocado, broccoli, chickpeas, coffee, cranberries, fermented dairy products, dietary fiber, green tea, soy, and whole grains are classified as beneficial components, while red meat, processed meat, refined grains, and high-fat diets (≥40% of energy from fat) are considered detrimental components (9). Dietary components are categorized based on sex-specific medians: for beneficial components, intake equal to or above the median scores 1 point, while intake below the median scores 0 points; for detrimental components, intake below the median scores 1 point, while intake equal to or above the median scores 0 points. The total score is the sum of all component scores, ranging from 0 to 13, with higher scores indicating dietary patterns more favorable to gut microbiota health (16).

To control for confounding effects, we included a range of covariates in the analysis. Demographic covariates included age (as a continuous variable in years), gender (male or female), and race/ethnicity (Non-Hispanic White, Non-Hispanic Black, Mexican American, and other racial/ethnic groups). Socioeconomic covariates included the poverty income ratio (PIR, a continuous variable), educational level (categorized as less than high school, high school or GED, and above high school), and marital status (married/living with partner, single, or never married). Lifestyle factors included smoking status (categorized as never smoker, former smoker, and current smoker) and alcohol consumption (categorized as never drinker, low drinker, and heavy drinker). Health-related covariates included hypertension status (yes or no), body mass index (BMI, as a continuous variable in kg/m^2^), serum calcium level (as a continuous variable in mg/dL), and estimated glomerular filtration rate (eGFR, as a continuous variable in mL/min/1.73 m^2^). Additionally, diabetes status was included as a clinical covariate, categorized as diabetic, prediabetic, or non-diabetic. These covariates were selected based on their established association with kidney stone formation and gut microbiota health and were included in the multivariable models to minimize confounding bias.

In this study, diabetes was defined by the following criteria: fasting plasma glucose (FPG) ≥7.0 mmol/L (126 mg/dL) or HbA1c ≥6.5% (24). Additionally, the response to the question “Has a doctor ever diagnosed you with diabetes?” was used to confirm diabetes status, with “yes” indicating diabetes and “no” indicating non-diabetes. In Table 1, we categorized diabetes status into three groups: “diabetic,” “prediabetic,” and “non-diabetic.” For statistical analyses in other figures, the “prediabetic” and “non-diabetic” groups were combined to facilitate the assessment of the relationship between diabetes, DI-GM index, and KS.

**Table 1:** Baseline characteristics of the study participants.

Characteristics	KS (*N* = 19,841)		*p*-value
	Yes	No	
	(*N* = 1,912)	(*N* = 1,7,929)	
Gender (*n*/%)			<0.001
Male	1,056 (55.23)	8,905 (49.67)	
Female	856 (44.77)	9,024 (50.33)	
Race (*n*/%)			<0.001
Mexican American	239 (12.50)	2,583 (14.41)	
Other Hispanic	200 (10.46)	1,746 (9.74)	
Non-Hispanic Black	1,063 (55.60)	7,513 (41.90)	
Non-Hispanic White	238 (12.45)	3,749 (20.91)	
Other race	172 (9.00)	2,338 (13.04)	
Marital status (*n*/%)			<0.001
Married or living with a partner	1,210 (63.28)	10,626 (59.27)	
Single (widowed/divorced/separated)	503 (26.31)	3,830 (21.36)	
Never married	199 (10.41)	3,473 (19.37)	
Education (*n*/%)			0.152
Less than high school	437 (22.86)	3,852 (21.48)	
High school or GED	405 (21.18)	4,105 (22.90)	
Above high school	1,070 (55.96)	9,972 (55.62)	
Diabetes mellitus (*n*/%)			<0.001
Yes	497 (25.99)	2,673 (14.91)	
Pre-diabetes	410 (21.44)	3,952 (22.04)	
No	1,005 (52.56)	11,304 (63.05)	
Hypertension (*n*/%)			<0.001
Yes	952 (49.79)	6,180 (34.47)	
No	960 (50.21)	11,749 (65.53)	
Smoking status (*n*/%)			<0.001
Never smoker	909 (47.54)	10,007 (55.84)	
Former smoker	610 (31.90)	4,287 (23.92)	
Current smoker	393 (20.55)	3,626 (20.23)	
Drinking status (*n*/%)			<0.001
Never drinker	562 (29.39)	4,407 (24.58)	
Low drinker	207 (10.83)	1,561 (8.71)	
Heavy drinker	1,143 (59.78)	11,961 (66.71)	<0.001
Age (years), mean ± SD	55.12 ± 16.24	48.53 ± 17.51	<0.001
PIR, mean ± SD	2.50 ± 1.62	2.50 ± 1.63	0.773
BMI (kg/m^2^), mean ± SD	30.57 ± 6.87	29.16 ± 6.98	<0.001
Serum calcium (mg/dL), mean ± SD	9.37 ± 0.39	9.39 ± 0.36	<0.001
eGRF (mL/min/1.73 m^2^), mean ± SD	93.74 ± 16.27	99.47 ± 16.64	<0.001
DI-GM, mean ± SD	4.82 ± 1.65	4.94 ± 1.70	0.007
Beneficial to gut microbiota, mean ± SD	2.23 ± 1.41	2.31 ± 1.44	0.033
Unbeneficial to gut microbiota, mean ± SD	2.59 ± 1.07	2.62 ± 1.05	0.175

BMI, body mass index; PIR, poverty income ratio; eGRF, estimated glomerular filtration rates; KS, kidney stone; DI-GM, dietary index for gut microbiota.

### 2.5 Eligibility criteria

#### 2.5.1 Inclusion criteria

Participants with complete records in the NHANES database and valid KS status and DI-GM scores.

#### 2.5.2 Exclusion criteria

Individuals with unknown KS status or incomplete data (e.g., missing BMI or PIR).

### 2.6 Sample size determination and sampling procedures

NHANES employed a stratified, multistage probability sampling design to ensure data representativeness and accuracy. The final sample size comprised 19,841 participants.

### 2.7 Data collection tools and procedures

Data collection was conducted through standardized questionnaires (household interviews) and physical examinations at mobile examination centers. Dietary data were collected using 24-h dietary recall and were used to calculate DI-GM scores.

### 2.8 Data quality control methods

Data were collected by NHANES staff and subjected to quality control procedures to ensure accuracy. All measurements were calibrated and validated using standardized protocols.

### 2.9 Data entry and analysis

**Figure 1:**
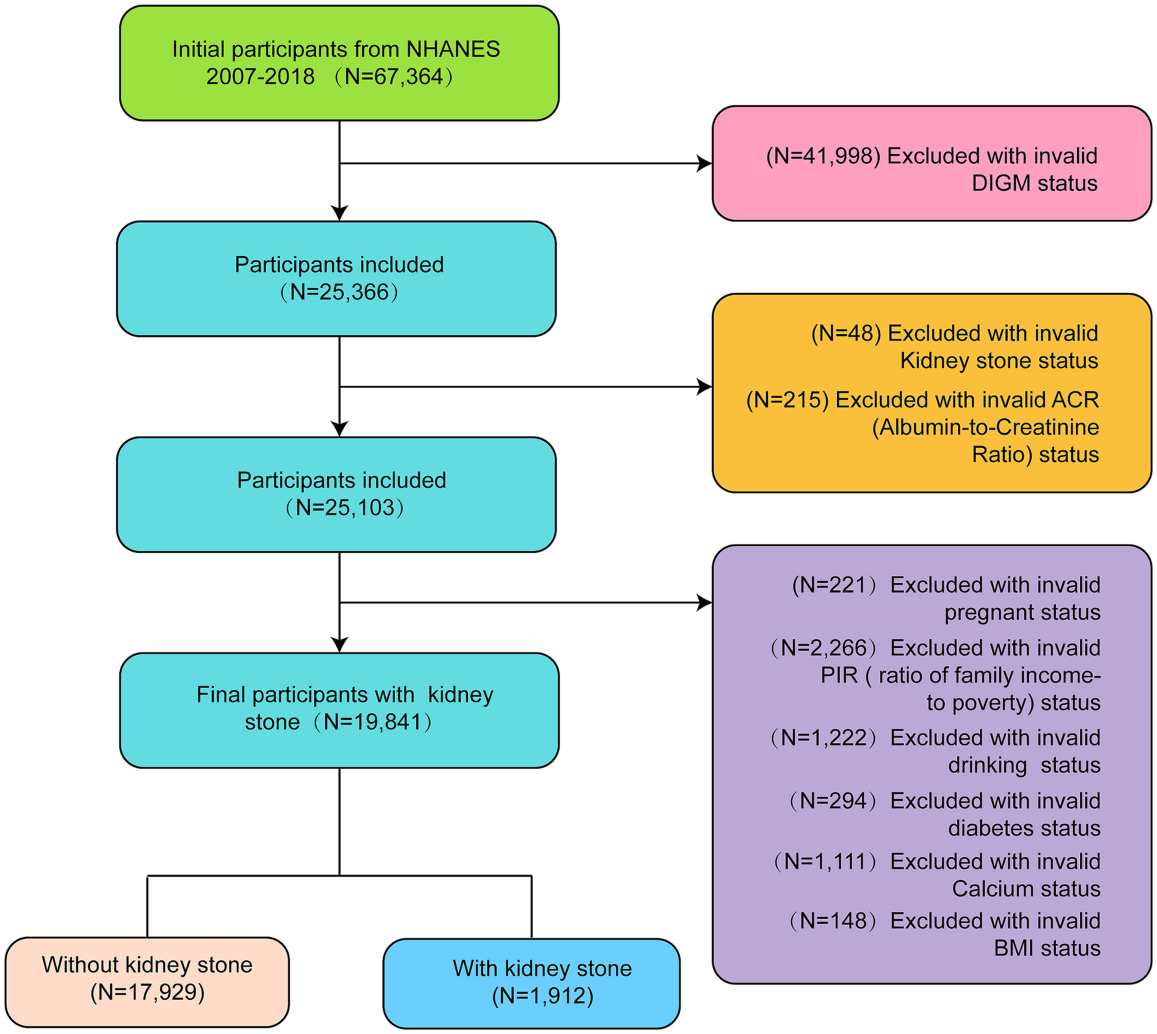
Flowchart of the selection of participants from NHANES 2007–2018 based on DI-GM (dietary index for gut microbiota).

Statistical analyses in this study followed CDC guidelines. Continuous variables are expressed as mean ± standard deviation and categorical variables as percentages. Normal data were analyzed by ANOVA, non-normal data by the Kruskal–Wallis test, and categorical data by chi-square test. A multivariable logistic regression model evaluated the association between DI-GM and KS, providing odds ratio (OR) with 95% confidence intervals (95% CI). Model 1 had no covariate adjustments; Model 2 adjusted for sex, age, and race; Model 3 further adjusted for education, marital status, smoking, drinking, hypertension, PIR, calcium, eGFR, and BMI. Subgroup analyses used multivariable regression, and mediation analysis assessed diabetes as a mediator for DI-GM and KS. A *p*-value <0.05 was considered significant. Analyses were conducted using Empower version 4.2 (X&Y Solutions, Inc., Boston, MA, United States) and R version 3.4.3 (R Foundation) (see Figure 1).

### 2.10 Ethical consideration

All NHANES data were approved by the NCHS Research Ethics Review Board, and all participants signed informed consent forms. The data are publicly available and do not involve personal privacy or additional ethical approval.

## 3 Results

### 3.1 Characteristics of the study population

Of the 19,841 participants analyzed, 1,912 (9.64%) were identified with KS. A distinct profile emerged for those with KS compared to those without. Males were more frequently affected (55.23%) than females, with significant racial differences observed, as the prevalence of KS was higher among non-Hispanic Black individuals. Marital status was also associated with KS presence, with a larger proportion of affected participants being married or living with a partner. Regarding health conditions, those with KS had higher rates of diabetes mellitus (25.99%) and hypertension (49.79%). Lifestyle factors also varied, with a higher proportion of KS patients being current smokers (20.55%) and heavy drinkers (59.78%). Clinically, participants with KS were older on average (55.12 ± 16.24 years) and had a higher mean BMI (30.57 ± 6.87 kg/m^2^) compared to those without KS. Moreover, mean serum calcium levels were lower, while estimated glomerular filtration rates (eGFR) were also reduced among participants with KS (93.74 ± 16.27 mL/min/1.73 m^2^). Analysis of dietary indices related to gut microbiota revealed a slightly lower mean score in the “beneficial to gut microbiota” category among those with KS (2.23 ± 1.41), though no significant differences were found in the “Unbeneficial to gut microbiota” category (see Table 1).

### 3.2 Logistic regression analyses

Table 2 revealed a statistically significant association between the DI-GM and KS prevalence, even after adjusting for various confounding factors. Participants with a DI-GM score of 6 or higher had a 17% lower prevalence of KS compared to those with a score of 0–3 (Model 3: OR = 0.83, 95% CI: 0.72–0.95, *p* = 0.007), with a significant trend of decreasing KS risk as DI-GM scores increased (*p*-trend <0.001). The “beneficial to gut microbiota” component of DI-GM was also independently associated with a lower KS risk (Model 3: OR = 0.94, 95% CI: 0.90–0.97, *p* < 0.001). Additionally, the analysis indicated that diabetes was associated with a higher prevalence of KS, with diabetic individuals having a 46% higher likelihood of developing KS compared to non-diabetic participants (Model 3: OR = 1.46, 95% CI: 1.29–1.65, *p* < 0.001).

**Table 2:** Odds ratio and 95% confidence intervals for KS according to DI-GM and diabetes.

Characteristics	Model 1		Model 2		Model 3	
	OR (95% CI)	*p*-value	OR (95% CI)	*p*-value	OR (95% CI)	*p*-value
DI-GM	0.96 (0.93, 0.99)	0.005	0.94 (0.91, 0.97)	<0.001	0.95 (0.92, 0.98)	<0.001
0–3	Ref		Ref		Ref	
4	0.96 (0.83, 1.11)	0.603	0.95 (0.82, 1.09)	0.444	0.94 (0.82, 1.09)	0.443
5	0.91 (0.79, 1.05)	0.216	0.88 (0.76, 1.02)	0.092	0.90 (0.78, 1.04)	0.159
≥6	0.87 (0.76, 0.99)	0.035	0.79 (0.76, 0.99)	0.035	0.83 (0.72, 0.95)	0.007
*p*-trend		<0.001		<0.001		<0.001
Beneficial to gut microbiota	0.96 (0.93, 0.99)	0.019	0.93 (0.90, 0.97)	<0.001	0.94 (0.90, 0.97)	<0.001
Unfavorable to gut microbiota	0.97 (0.93, 1.01)	0.181	0.96 (0.92, 1.01)	0.084	0.99 (0.95, 1.04)	0.657
Diabetes						
No	Ref		Ref		Ref	
Yes	2.00 (1.80, 2.24)	<0.001	1.70 (1.51, 1.91)	<0.001	1.46 (1.29, 1.65)	<0.001

CI, confidence interval; KS, kidney stone; DI-GM, dietary index for gut microbiota; NHANES, National Health and Nutrition Examination Survey; OR, odds ratio; PIR, poverty income ratio; eGFR, estimated glomerular filtration rate. In sensitivity analysis, DI-GM was converted from a continuous variable to a categorical variable (0–3, 4, 5, ≥6).

Table 3 shows that both the “beneficial to gut microbiota” and “unfavorable to gut microbiota” components of the DI-GM were significantly associated with diabetes prevalence. Specifically, the “beneficial to gut microbiota” component showed a borderline association with reduced diabetes risk after full adjustment for confounding factors (Model 3: OR = 0.97, 95% CI: 0.94–1.00, *p* = 0.0285). The “unfavorable to gut microbiota” component demonstrated a stronger inverse association, where a higher score was associated with a significantly lower diabetes prevalence (Model 3: OR = 0.90, 95% CI: 0.86–0.93, *p* < 0.0001).

**Table 3:** Association between DI-GM and diabetes of the NHANES 2007–2018 participants.

Characteristics	Model 1		Model 2		Model 3	
	OR (95% CI)	*p*-value	OR (95% CI)	*p*-value	OR (95% CI)	*p*-value
DI-GM	0.92 (0.90, 0.95)	<0.0001	0.91 (0.89, 0.93)	<0.0001	0.93 (0.91, 0.96)	<0.0001
0–3	Ref		Ref		Ref	
4	0.97 (0.87, 1.09)	0.6152	0.96 (0.85, 1.08)	0.5128	0.99 (0.88, 1.13)	0.9084
5	0.80 (0.71, 0.89)	0.6152	0.78 (0.69, 0.88)	<0.0001	0.81 (0.71, 0.92)	0.0012
≥6	0.74 (0.67, 0.82)	<0.0001	0.68 (0.61, 0.77)	<0.0001	0.76 (0.68, 0.86)	0.0012
*p*-trend		<0.001		<0.001		<0.001
Beneficial to gut microbiota	0.93 (0.90, 0.95)	<0.0001	0.95 (0.92, 0.98)	0.0006	0.97 (0.94, 1.00)	0.0285
Unfavorable to gut microbiota	0.94 (0.91, 0.98)	0.0014	0.86 (0.83, 0.89)	<0.0001	0.90 (0.86, 0.93)	<0.0001

CI, confidence interval; DI-GM, dietary index for gut microbiota; NHANES, National Health and Nutrition Examination Survey; OR, odds ratio; PIR, poverty income ratio; eGFR, estimated glomerular filtration rate. In sensitivity analysis, DI-GM was converted from a continuous variable to a categorical variable (0–3, 4, 5, ≥6).

**Figure 2:**
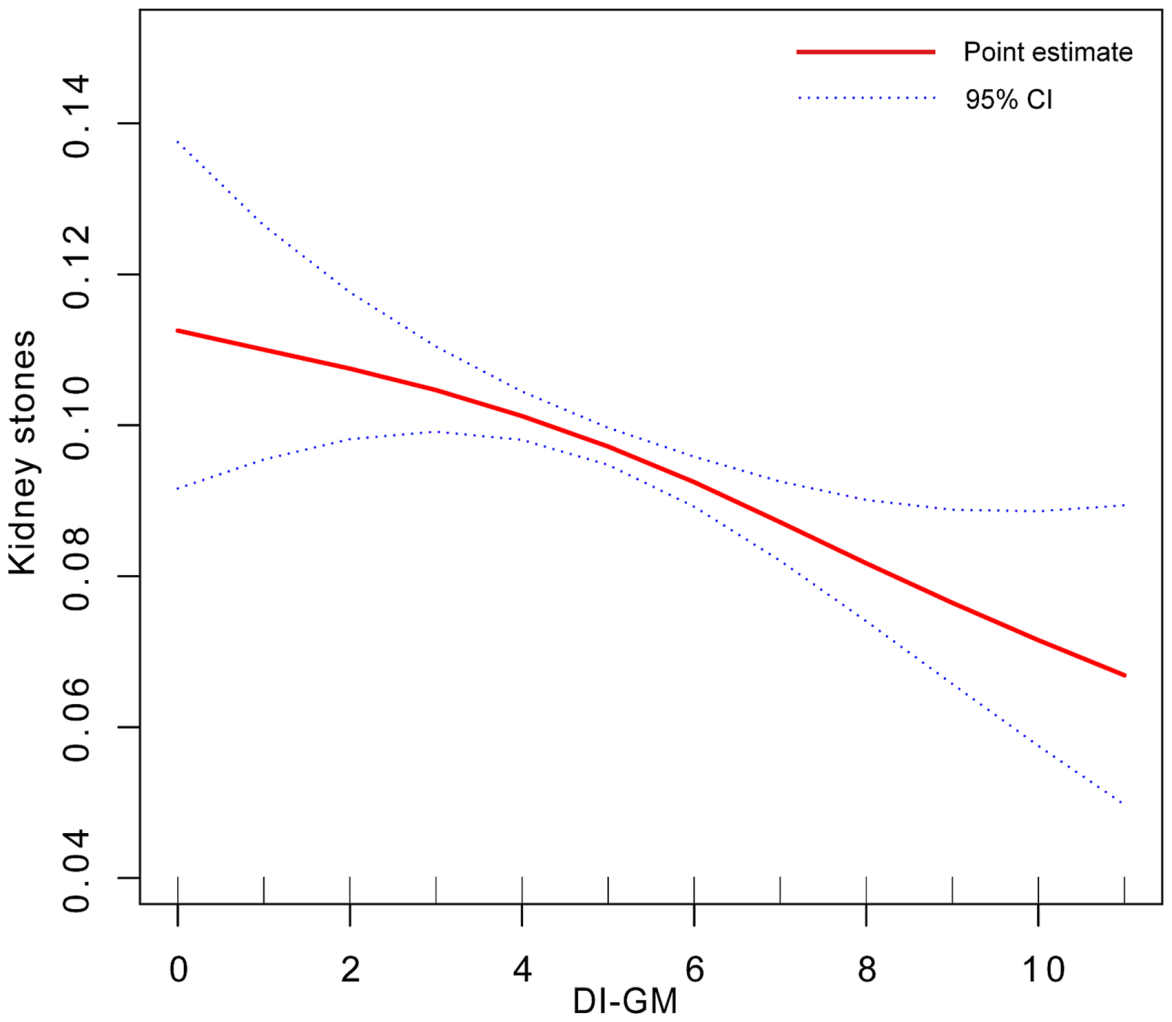
Dose-response relationship between DI-GM and KS. Adjusted for gender + race + age + education level + marital status + smoking status + drinking status + hypertension + PIR + serum calcium + eGRF + BMI.

The results of the RCS demonstrated a nonlinear correlation between DI-GM and the prevalence of KS (Figure 2).

### 3.3 Subgroup analysis

**Figure 3:**
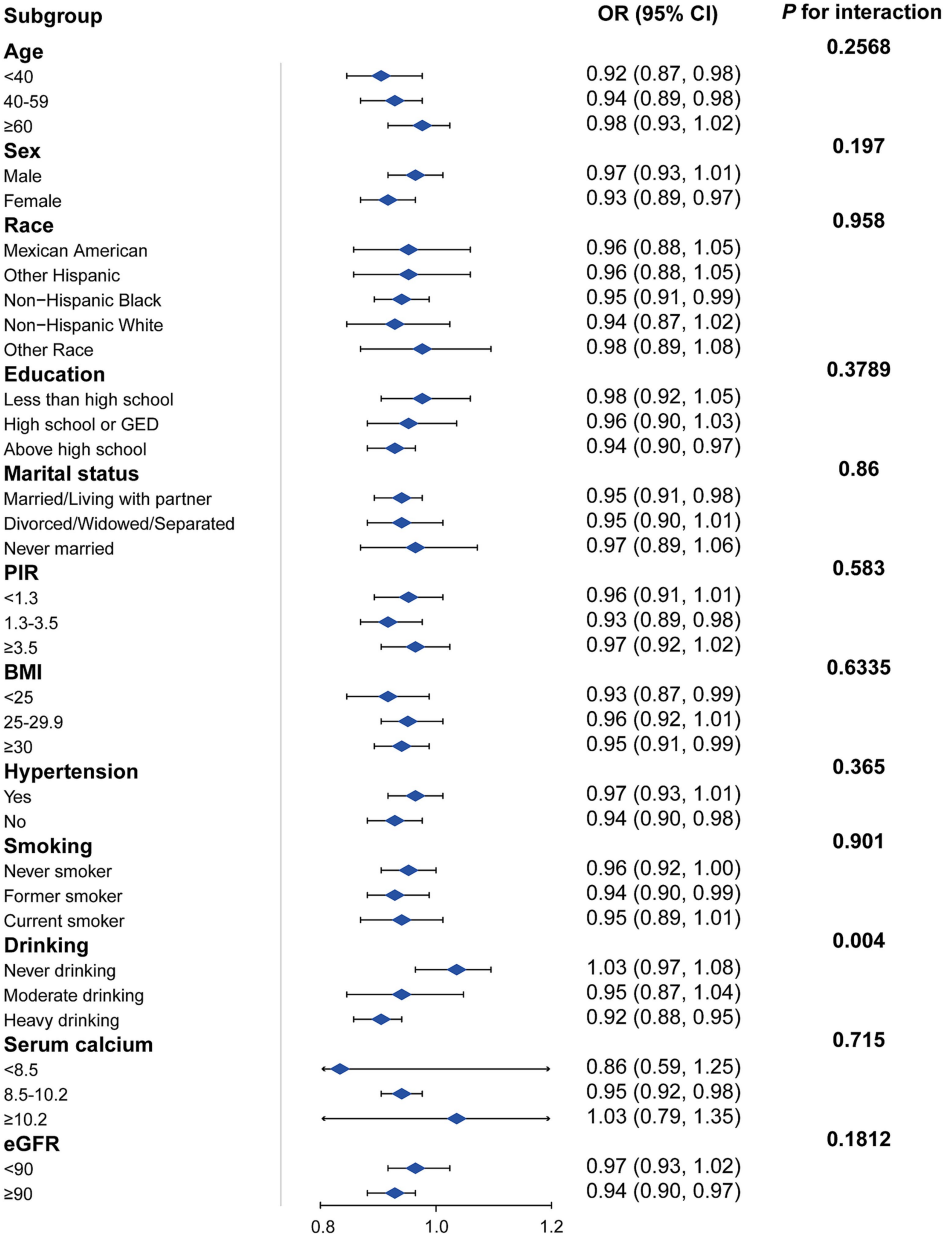
Subgroup analysis of the association between DI-GM and KS. Adjusted for age + sex + race + education + marital status + poverty income ratio + hypertension + BMI + smoking status+ drinking + serum calcium + eGFR. The strata variable was not included when stratifying by itself. PIR, poverty income ratio; eGRF, estimated glomerular filtration rates; DI-GM, dietary index for gut microbiota, BMI, body mass index.

Figure 3 presents a subgroup analysis of the association between DI-GM scores and KS prevalence across various demographic and health-related factors. The analysis shows that the protective association between DI-GM and KS is consistent across most subgroups, including age, sex, race, education, marital status, BMI, hypertension status, serum calcium levels, and eGFR categories (*p* for interaction >0.05). Drinking status is the only factor that significantly modified the association (*p* = 0.004). Among heavy drinkers, a higher DI-GM score was associated with a reduced risk of KS (OR = 0.92, 95% CI: 0.88–0.95), while no significant association was observed between never-drinkers or moderate-drinkers.

### 3.4 Results of mediation analysis

**Figure 4:**
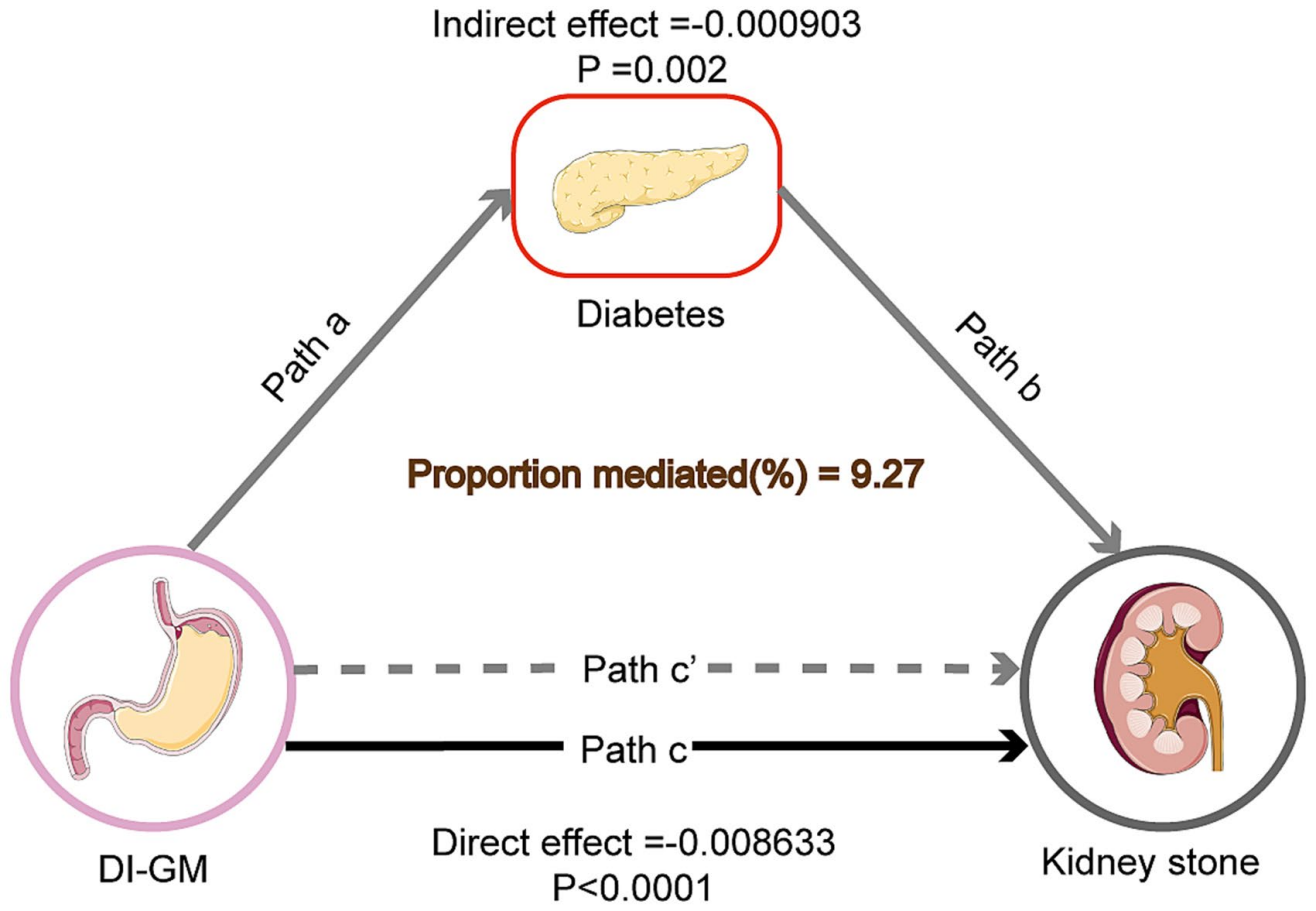
Mediation model of the effect of DI-GM on KS risk through diabetes. Solid arrows represent direct effects, while dashed arrows represent indirect effects through diabetes. Path coefficients (*β*) are shown along each arrow, indicating the strength of the relationship. DI-GM refers to the dietary index for gut microbiota, diabetes is the mediator, and KS risk is the outcome. The mediation effect of diabetes accounts for 9.27% of the total effect of DI-GM on KS risk.

The outcome of the mediation analysis shows that diabetes partially mediates the relationship between DI-GM and KS prevalence. In Model 3, the total effect of DI-GM on KS was significant (Estimate = −0.009489, *p* < 0.0001). The mediation effect through diabetes was also significant (Estimate = −0.000903, *p* = 0.0020), accounting for 9.27% of the total effect. The direct effect of DI-GM remained significant after adjusting for diabetes (Estimate = −0.008633, *p* < 0.0001) (see Table 4 and Figure 4).

**Table 4:** Involvement of diabetes in mediating the relationship between DI-GM and KS.

Characteristics	Model 1		Model 2		Model 3	
	Estimate	*p*-value	Estimate	*p*-value	Estimate	*p*-value
Total effect	−0.007066	0.0020	−0.011120	<0.0001	−0.009489	<0.0001
Mediation effect	−0.001535	<0.0001	−0.001552	<0.0001	−0.000903	0.0020
Direct effect	−0.005589	0.0180	−0.009662	<0.0001	−0.008633	<0.0001
Proportion mediated	21.31%	0.0020	13.54%	<0.0001	9.27%	0.0020

CI, confidence interval; DI-GM, the dietary index for gut microbiota; PIR, poverty income ratio; eGFR, estimated glomerular filtration rate.

## 4 Discussion

This study, based on NHANES data (2007–2018), provides robust evidence linking the DI-GM to KS prevalence. Higher DI-GM scores, reflective of diets rich in fiber, whole grains, and fermented foods, were associated with a reduced risk of KS, even after adjusting for multiple confounders. Furthermore, diabetes was found to partially mediate this relationship, accounting for 9.27% of the total effect. These findings emphasize the critical role of dietary patterns that support gut microbiota health in preventing KS, highlighting an avenue for non-invasive, dietary-based preventive strategies.

### 4.1 Comparison with previous studies

This study contributes to a growing body of research exploring the role of gut microbiota in KS pathogenesis. Prior studies have highlighted the role of specific microbial taxa, such as *Oxalobacter formigenes*, in degrading oxalates, reducing urinary oxalate excretion, and lowering the risk of calcium oxalate stones (25). Our findings align with this mechanism but extend the scope by demonstrating the impact of overall dietary patterns on gut microbial functionality. Furthermore, while previous research has identified reduced urinary citrate as a contributing factor to KS formation, this study links gut microbiota dysbiosis, such as an increased abundance of *Escherichia-Shigella*, to lower citrate levels, suggesting a novel pathway connecting microbiota composition with lithogenesis (26). Unlike studies that focus on single microbial or dietary factors, this research underscores the cumulative impact of diet-induced microbial shifts on KS risk (27, 28).

### 4.2 Subgroup-specific effects

The protective effect of DI-GM appeared more pronounced in subgroups such as females, non-Hispanic Black individuals, and heavy drinkers. Among heavy drinkers, the strong inverse association suggests that dietary components beneficial to gut microbiota may mitigate the adverse metabolic effects of alcohol on the kidneys. Alcohol consumption is known to disrupt gut microbiota balance, increasing intestinal permeability and systemic inflammation (29, 30). Diets rich in fiber and polyphenols, reflected in higher DI-GM scores, may counteract these disruptions by enhancing microbial diversity and short-chain fatty acid (SCFA) production, which have anti-inflammatory and renal-protective effects (31, 32). However, the absence of significant effects in non-drinkers and moderate drinkers highlights the complexity of interactions between alcohol, diet, and gut microbiota. These findings align with prior studies on alcohol’s effects on gut health and suggest the need for further investigation into subgroup-specific dietary interventions (33).

### 4.3 Mechanistic insights

Gut microbiota impacts KS formation through several pathways. First, oxalate metabolism remains a central mechanism, as oxalate-degrading bacteria, such as *Oxalobacter formigenes*, reduce hyperoxaluria by breaking down dietary oxalate in the gut and limiting its intestinal absorption, which is a key risk factor for calcium oxalate stones (34, 35). Loss of these bacteria due to dysbiosis or antibiotic use is associated with increased urinary oxalate excretion and higher KS risk (36, 37). Second, microbial metabolites, such as short-chain fatty acids (SCFAs), including butyrate and acetate, not only improve gut barrier function but also modulate systemic inflammation and oxidative stress, both of which are critical in maintaining renal health and reducing stone risk (31, 38). Moreover, SCFAs help regulate metabolic pathways related to citrate excretion and urinary pH, creating a less favorable environment for stone formation (39). Lastly, gut dysbiosis promotes systemic inflammation by increasing gut permeability and endotoxin levels, such as lipopolysaccharides (LPS), impairing renal function and exacerbating metabolic conditions (22, 40). These mechanisms highlight the potential of dietary patterns that enhance gut microbial diversity and functionality to protect against KS.

### 4.4 Diabetes as a mediator

The mediation analysis revealed that diabetes accounted for 9.27% of the total effect of DI-GM on KS risk. While this proportion is modest, it underscores the interconnected roles of diet, gut microbiota, and metabolic health. Diabetes exacerbates lithogenesis through hyperglycemia-induced increases in urinary calcium and reduced citrate excretion (41, 42). These mechanisms create a urinary environment conducive to stone formation, with higher calcium concentrations promoting calcium oxalate crystal aggregation and reduced citrate levels decreasing the inhibition of such crystallization. Furthermore, diabetic patients often exhibit changes in urinary pH due to impaired renal ammoniagenesis, which may further contribute to stone formation by facilitating uric acid stone precipitation (43).

In addition to metabolic alterations, diabetic patients have distinct gut microbial profiles characterized by reduced microbial diversity and an increased abundance of pro-inflammatory taxa, which exacerbate systemic inflammation and oxidative stress (44, 45). This gut dysbiosis has downstream effects on metabolic pathways relevant to kidney stone formation, such as increased intestinal permeability and the translocation of microbial endotoxins like lipopolysaccharides (LPS) into circulation. LPS triggers systemic inflammation, which has been linked to renal tubular damage, altered calcium handling, and increased oxalate excretion, thereby enhancing lithogenesis. Moreover, inflammation may directly disrupt gut-kidney axis interactions, highlighting the multifaceted role of gut microbiota in kidney stone risk (46, 47).

These findings align with prior studies demonstrating that high-fiber, low-sugar diets support beneficial bacterial taxa, such as *Bifidobacterium* and *Lactobacillus*, which improve insulin sensitivity and reduce urinary calcium excretion (21, 48). Additionally, such dietary patterns promote the production of short-chain fatty acids (SCFAs), such as butyrate, which not only improve gut barrier integrity but also have anti-inflammatory properties that may mitigate the systemic effects of diabetes on kidney stone formation. These dietary influences extend beyond diabetes itself to directly impact kidney stone risk through gut microbiota-mediated pathways (49, 50).

The direct effect of DI-GM on kidney stones, which constitutes a larger proportion of the total effect, highlights the importance of dietary quality in directly modulating gut microbiota composition and kidney stone risk, independent of diabetes.

### 4.5 Strengths, limitations, and future directions

This study’s strengths include its use of a large, nationally representative dataset and robust statistical analyses, which enhance its reliability and generalizability within the U.S. population. However, several limitations must be acknowledged. First, while the DI-GM is a validated tool for assessing the impact of diet on gut microbiota, it provides an indirect estimation rather than a direct measurement of microbial composition, highlighting the need for future studies to incorporate microbiome sequencing technologies. Second, the NHANES dataset lacks detailed information on specific kidney stone types (e.g., calcium oxalate, uric acid stones), comprehensive antibiotic use data (e.g., frequency, duration, and type), and urinary factors critical to kidney stone risks, such as oxalate and citrate levels. These urinary metabolites play essential roles in kidney stone formation: oxalate is a key component of calcium oxalate stones, while citrate acts as an inhibitor of crystal aggregation. The absence of such data limits the ability to fully assess the mechanistic pathways linking diet, gut microbiota, and kidney stone risk. Antibiotic use, in particular, is known to disrupt gut microbial taxa, including oxalate-degrading bacteria, potentially influencing stone risk. Lastly, while diabetes was included as a mediating variable, future research should explore additional mediators, such as systemic inflammation, oxalate metabolism, and other urinary factors, to better understand the complex pathways linking diet, gut microbiota, and kidney stones. Future studies should also examine diverse populations and leverage multi-omics approaches to provide a more nuanced understanding of these relationships.

### 4.6 Practical implications and conclusion

Higher DI-GM scores, reflecting diets rich in fiber, whole grains, fruits, vegetables, and fermented foods, are associated with a reduced risk of KS, partially mediated by diabetes. These findings highlight the importance of dietary strategies targeting gut microbiota health in KS prevention. Specifically, increasing the intake of fiber-rich foods (e.g., legumes, whole grains, leafy greens), reducing added sugars and processed foods, and incorporating probiotics or fermented foods (e.g., yogurt, kefir, sauerkraut) may enhance gut microbial diversity and functionality (51). For high-risk populations, such as individuals with diabetes or recurrent KS, targeted interventions like personalized nutrition plans and supplementation with oxalate-degrading probiotics (*Oxalobacter formigenes*) warrant further exploration (52). Future research should focus on developing practical dietary recommendations and microbial therapies to address KS risk effectively.

## 5 Conclusion

This study provides compelling evidence that dietary patterns promoting gut microbiota health, as reflected by higher DI-GM scores, are associated with a reduced risk of KS. Compared to prior research focusing on individual dietary components or microbial taxa, this study emphasizes the broader impact of cumulative dietary patterns on gut microbiota and metabolic health. These findings suggest that promoting diets aligned with higher DI-GM scores could serve as a cost-effective, non-invasive strategy to prevent KS, particularly in populations at high risk for diabetes and metabolic disorders. Future research should focus on translating these findings into practical dietary recommendations and developing targeted interventions to address this significant public health challenge.

## Data Availability

The raw data supporting the conclusions of this article will be made available by the authors, without undue reservation.
